# Optimization of Spinal Cord Perfusion Pressure for Improvement of Neurological Outcome in Traumatic Spinal Cord Injury: A Systematic Review and Meta-Analysis

**DOI:** 10.1177/2689288X251377015

**Published:** 2025-09-12

**Authors:** Gabrielle E.A. Hovis, Brandon Sherrod, Nitin Agarwal, Berje H. Shammassian, Anthony M. DiGiorgio, Ramesh Grandhi, Bryce Picton

**Affiliations:** ^1^ Department of Neurosurgery, University of California, Irvine, Irvine, California, USA.; ^2^ Department of Neurosurgery, Clinical Neurosciences Center, University of Utah, Salt Lake City, Utah, USA.; ^3^ Department of Neurological Surgery, University of Pittsburgh Medical Center, Pittsburgh, Pennsylvania, USA.; ^4^ Department of Neurological Surgery, Louisiana State University Health, New Orleans, Louisiana, USA.; ^5^ Department of Neurological Surgery, University of California, San Francisco, San Francisco, California, USA.

**Keywords:** cerebrospinal fluid drain, spinal cord perfusion pressure, thoracoabdominal aortic aneurysm, traumatic spinal cord injury

## Abstract

The optimal hemodynamic management of traumatic spinal cord injury (tSCI) is not well established. We performed a systematic review and meta-analysis of patients with acute tSCI to assess the role of lumbar cerebrospinal fluid drainage (CSFD) and to identify factors predictive of neurological improvement. Three studies involving 46 patients with acute tSCI, lumbar drain placement with CSFD, and available pre- and post-intervention American Spinal Injury Association (ASIA) Impairment Scale or International Standards for Neurological Classification of Spinal Cord Injury (ISNCSCI) motor scores were identified. One study was analyzed separately (Cohort A) using ASIA grade because post-intervention ISNCSCI motor scores were not reported. In the remaining two studies (Cohort B), two independent meta-analyses with meta-regressions were performed to determine the mean difference in ISNCSCI motor scores by CSFD and time to decompression. Individual patient data were used for all analyses. In Cohort A, there was no significant difference in ASIA grade before lumbar CSFD and at the last follow-up (Wilcoxon signed rank test: *p* = 0.130). In Cohort B, female sex and anterior decompression were associated with greater neurological recovery compared with male sex (*p* = 0.045) and a combined approach to decompression (*p* = 0.048), respectively. There were no other significant indicators of better motor scores in either cohort. CSFD with a target intrathecal pressure, rather than volume restrictions, may allow for sufficient fluid drainage to maximize spinal cord perfusion pressure (SCPP). Patients may benefit most from individualized management of SCPP because of the wide variance in interpatient and chronological intrathecal pressure.

## Introduction

In the United States, the annual incidence of traumatic spinal cord injury (tSCI) is approximately 90,000, with an age-standardized rate of 26 per 100,000 population.^1^ Spinal cord ischemia is a known mechanism of secondary injury after tSCI,^2–5^ but strategies for prevention and management are controversial. Traditionally, tSCI management has focused on maintaining mean arterial pressure (MAP) to promote blood flow to the spinal cord. However, studies on outcomes relating to augmented MAP goals have yielded inconsistent results, and this hemodynamic parameter only serves as a surrogate marker for actual spinal cord perfusion.^6,7^

The concept of directly monitoring and optimizing spinal cord perfusion pressure (SCPP) emerged over a decade ago, inspired by the analogous standard of cerebral perfusion pressure monitoring in traumatic brain injury.^8^ Two important measurements in the assessment of SCPP are cerebrospinal fluid pressure (CSFP), measured in the lumbar cistern, and intraspinal pressure (ISP) at the site of injury.^9^

Recent studies have shown the superiority of SCPP over MAP as an independent predictor of neurological recovery after tSCI and that monitoring and maintenance of SCPP, rather than a sole focus on MAP goals, may improve patient outcomes.^3,7,10^ Because SCPP monitoring is not widely practiced and the current quality of evidence supporting SCPP management is low, no guideline recommendation has been made.^6^

Lumbar drain (LD) placement and intraoperative cerebrospinal fluid drainage (CSFD) with a target CSFP during thoracoabdominal aortic aneurysm repair have been shown to effectively reduce paraplegia and other neurological complications in both open^11–14^ and endovascular^15,16^ thoracoabdominal aortic aneurysm repair. However, there is limited evidence supporting the use of LDs to reduce CSFP and improve spinal cord perfusion in tSCI, despite the similar pathophysiology.^17^

We conducted a systematic review and meta-analysis with individual participant data on the utility of lumbar catheter placement for CSFD to improve neurological outcomes in acute tSCI.

## Materials and Methods

**FIG. 1.**
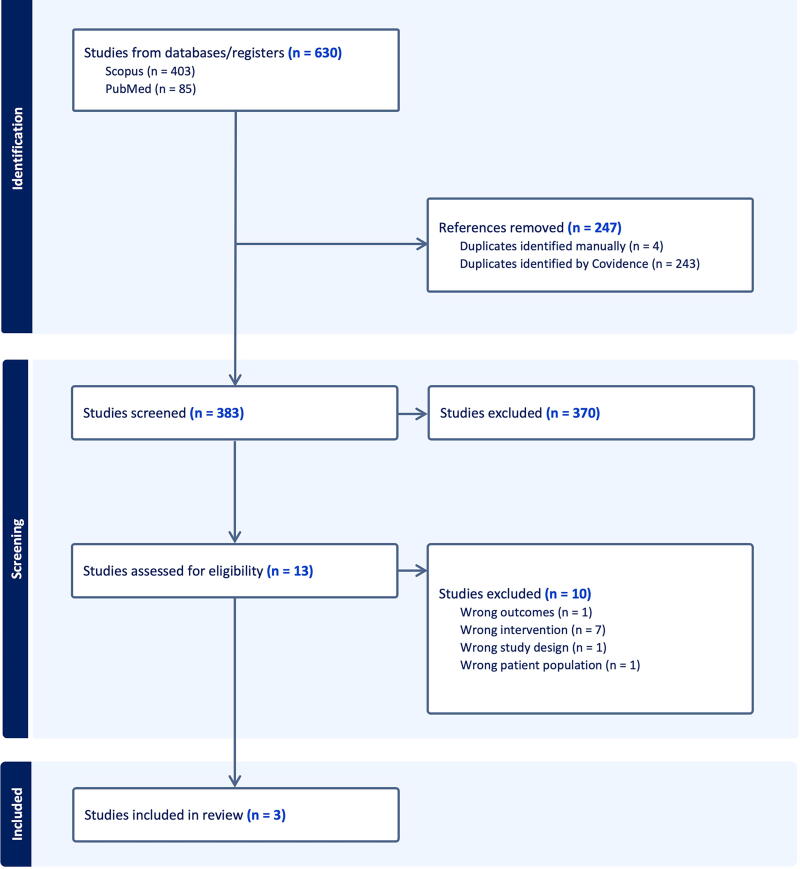
PRISMA diagram detailing study inclusion and reasons for exclusion. Diagram derived from Covidence template. PRISMA, Preferred Reporting Items for Systematic Reviews and Meta-Analyses.

A systematic review was conducted according to Preferred Reporting Items for Systematic Reviews and Meta-Analyses (PRISMA) guidelines.^18^ Reports of LD placement for tSCI published before July 2024 were identified using the search terms “spinal cord injury,” “spinal cord infarct,” “spinal cord ischemia,” “lumbar drain,” “intrathecal catheter,” “lumbar catheter,” “intrathecal drain,” “CSF drain,” and “cerebrospinal fluid drain” alone and in combination. The search returned 383 unique studies across five databases (PubMed, MEDLINE, Scopus, Web of Science, and Cochrane). Using the Covidence systematic review software (Veritas Health Innovation, Melbourne, Australia), the literature was screened for the following inclusion criteria: (1) acute tSCI, (2) placement of lumbar catheter with CSFD, and (3) available American Spinal Injury Association (ASIA) Impairment Scale grade or International Standards for Neurological Classification of Spinal Cord Injury (ISNCSCI) motor scores. Studies with iatrogenic SCI, non-human subjects, pediatric or pregnant patients, inaccessible full-text articles, non-English text, book chapters, conference abstracts, reviews, systematic reviews, meta-analyses, case reports, or insufficient data were excluded; 3 articles met the criteria for analysis (Fig. 1). Among these, 2 studies were randomized controlled trials (RCTs), and the remaining study was an observational, nonrandomized clinical trial. Variables of interest included age, sex, CSFD, level of injury, initial ASIA grade, ASIA grade at last follow-up visit, initial ISNCSCI motor score, ISNCSCI motor score at last follow-up, duration of indwelling LD (hours), surgical approach (anterior, posterior, or combined decompression with or without fusion), length of stay (days), and follow-up period (months).

Because both ASIA and ISNCSCI motor scores were not available pre- and postoperatively for all three studies, the data were grouped accordingly for analysis. We divided the studies into 2 groups for independent analysis: Cohort A included the patients reported by Hogg et al.,^19^ and Cohort B included the patients reported by Kwon et al.^20^ and Theodore et al.^3^ All statistical analyses were performed in R (v2023.06, R Foundation for Statistical Computing, Vienna, Austria).^21^ Descriptive statistics were calculated for Cohort A, and Wilcoxon signed rank tests were used to compare baseline and follow-up ASIA grades.

For Cohort B, a meta-analysis was performed to compare the mean change in ISNCSCI motor scores from baseline to last follow-up by CSFD group. A separate meta-analysis of the mean change in ISNCSCI motor scores was performed based on time to decompression of <19 h (early) versus ≥19 h (late), an interval chosen based on the median time to decompression in Cohort B. A fixed-effects model was used because of minimal heterogeneity between the studies. LDs remained in place for 72 h in the study by Kwon et al.^20^ and 120 h in that of Theodore et al.^3^ Indwelling LD duration was divided into 2 groups using this division of Cohort B, and mean differences were calculated for each. The effect of indwelling LD duration on neurological function was assessed using the nonparametric Mann–Whitney U test. A multivariate linear regression was performed to identify factors predictive of an improvement in ISNCSCI motor score.

## Results

### Cohort A

Hogg et al.^19^ recruited patients with tSCI from the Injured Spinal Cord Pressure Evaluation trial (NCT02721615); criteria for the trial included severe tSCI (ASIA grades A–C), age 18–70 years, and surgery performed within 72 h of injury. The cohort comprised 13 patients with a mean age of 47 years and a mean follow-up of 6.5 months (Table 1). All patients underwent decompression with intraoperative intradural placement of a fiberoptic transducer (Codman Microsensor Transducer, DePuy Synthes, Leeds, UK), in addition to LD insertion with a pressure transducer for ISP monitoring. Multiple CSF samples were collected and analyzed for metabolites. The authors found that pressure and biochemical values from the injury site were significantly different from measurements collected from lumbar CSF. Intraspinal pressure and CSFP were also found to be variably correlated, suggesting a dynamic quality of cord compression.

**Table 1.** Patient Demographics, Intervention Characteristics, and Neurological Outcomes for Cohort A

Patient no.	Age (years)	Sex	Level	Initial ASIA	ASIA at last follow-up	Approach	Duration of monitoring (h)	Follow-up (months)
1	67	M	C3	A	A	P	104	1
2	67	M	C4	C	C	P	128	12
3	32	M	C4	C	D	C	151	10
4	39	M	T7	A	B	P	113	15
5	35	M	C4	C	A	C	85	12
6	27	M	L1	C	D	P	112	6
7	50	M	C5	B	B	P	139	2
8	47	M	T8	A	C	P	106	12
9	57	M	C4	A	A	P	147	3
10	66	M	C4	A	A	P	129	4
11	46	M	T12	A	C	P	52	4
12	52	M	C5	A	B	C	128	2
13	26	M	C6	A	B	C	71	1

Cohort A comprised patients from Hogg et al.^19^

ASIA, American Spinal Injury Association Impairment scale score; A, anterior decompression; C, combined anterior and posterior decompression; P, posterior decompression.

The cervical spine was the most common injury location (69.2%, 9 patients), the most common level being C4 in 5 (38.5%) patients. Injury at the thoracic level was reported in 3 (23.1%) patients and injury at the lumbar level in 1 patient (7.7%). The mean duration of indwelling LD was 113 h (range: 52–151). Posterior decompression was performed in 9 cases (69.2%) and a combined approach in 4 cases (30.8%). There was no significant difference in ASIA grade before lumbar CSFD and at the last follow-up (Wilcoxon signed rank test: *p* = 0.130).

### Cohort B

Kwon et al.^20^ performed a prospective RCT of patients with acute tSCI. All patients underwent lumbar intrathecal catheter insertion for 72 h and surgical decompression. Of the 22 included patients (mean age: 41.3 years), 11 were randomized to CSFD and 11 to the no-drainage group. CSFD did not result in significant CSFP reduction, but SCPP improved with spinal decompression.

Theodore et al.^3^ conducted a prospective RCT comparing MAP elevation to MAP augmentation with CSFD for acute cervical SCI within 24 h of presentation. All participants underwent lumbar catheter placement and decompressive surgery. Of the 11 included patients (mean age: 40.1 years), 4 were randomized to CSFD with MAP elevation to a CSFP goal of <10 mmHg for 5 days and 7 were randomized to MAP augmentation alone. CSFP was significantly lower and SCPP was significantly higher (*p* < 0.01) with CSFD relative to the control group. Four patients were lost to follow-up (3 from the control group and 1 from the experimental group), but among the remaining participants, motor scores improved from baseline at 180 days in both groups.

In combining the 2 trials, we analyzed a cohort of 33 patients with a mean age of 40.9 years (standard deviation [SD] = 12.4) that was 69.7% male (Table 2). The level of injury was most commonly cervical (84.8%), with the highest incidence of injury at C4 (33.3%). A thoracic injury level was recorded in 5 (15.2%) patients. The mean baseline ISNCSCI motor score was 25.2 (SD = 17.5), which improved to 43.2 (SD = 26.1) at last follow-up, with a mean improvement in motor score of 18.1 (SD = 22.0). The mean duration of LD monitoring was 88 h (SD = 23.0). A posterior approach to spinal cord decompression was used in 17 (51.5%) patients, an anterior decompression approach in 9 patients (27.3%), and a combined approach in 7 patients (21.2%). On multivariate linear regression, female patients had significantly greater improvement in ISNCSCI motor score relative to male patients (11.8 vs. 20.8, *p* = 0.045), and patients who underwent anterior decompression demonstrated a significant improvement in motor score relative to the combined approach (21.8 vs. 20.6, *p* = 0.048). Age, duration of indwelling LD, CSFD, and use of the posterior approach were not significantly predictive of ISNCSCI motor score improvement.

**Table 2.** Patient Demographics, Intervention Characteristics, and Total Motor Score in Cohort B by Study

Patient no.	Age (years)	Sex	Level	Duration of monitoring (h)	Time to decompression (h)	Approach	CSFD (Y/N)	Initial motor score	Motor score at last follow-up
Kwon et al. 2009^20^									
1	47	M	T3	72	37	P	Y	50	50
2	64	F	C5	72	26	P	Y	37	96
3	37	M	T8	72	22	P	Y	50	50
4	37	M	C5	72	16	P	Y	8	15
5	40	M	T3	72	23	P	Y	50	48
6	31	F	T9	72	42	P	Y	50	50
7	45	F	C6	72	23	A	Y	23	26
8	46	F	C4	72	12	A	Y	4	8
9	60	M	C5	72	19	P	Y	7	58
10	27	M	C4	72	14	A	Y	6	11
11	55	F	T9	72	24	P	Y	50	50
12	29	M	C6	72	10	A	N	57	98
13	34	M	C6	72	25	C	N	20	29
14	42	M	C6	72	21	P	N	20	24
15	46	F	C6	72	26	P	N	10	14
16	50	F	C5	72	26	C	N	9	13
17	23	M	C6	72	19	A	N	23	31
18	23	M	C6	72	11	P	N	25	28
19	30	M	C4	72	24	C	N	4	8
20	46	M	C5	72	33	A	N	16	70
21	31	M	C6	72	40	P	N	17	40
22	66	M	C4	72	20	P	N	20	44
Theodore et al. 2023^3^									
1	45	F	C4	120	5	P	Y	0	0
2	34	M	C4	120	14	C	Y	3	84
3	48	M	C4	120	24	P	Y	23	88
4	26	M	C4	120	18	A	Y	5	29
5	67	M	C4	120	9	P	N	38	41
6	47	M	C4	120	16	P	N	36	48
7	29	F	C5	120	5	C	N	38	56
8	50	M	C7	120	6	A	N	16	47
9	40	M	C6	120	9	C	N	32	58
10	28	F	C4	120	8	A	N	54	80
11	27	M	C5	120	16	C	N	32	34

CSFD, cerebrospinal fluid drainage; A, anterior decompression; C, combined anterior and posterior decompression; P, posterior decompression.

There was no significant difference in baseline neurological function between patients in the CSFD and no CSFD groups (*p* = 0.538) or between the groups with early or late decompression (*p* = 0.376). Baseline ISNCSCI motor scores were also similar between patients who had indwelling LDs for 72 versus 120 h (*p* > 0.999). The mean change in ISNCSCI motor scores was compared between an indwelling LD duration of 72 h (mean change = 13.9; SD = 19.4) and 120 h (mean change = 11.6; SD = 21.7). On nonparametric analysis, no difference was observed in the mean change in ISNCSCI motor score between the two groups (*p* = 0.325).

**FIG. 2.**
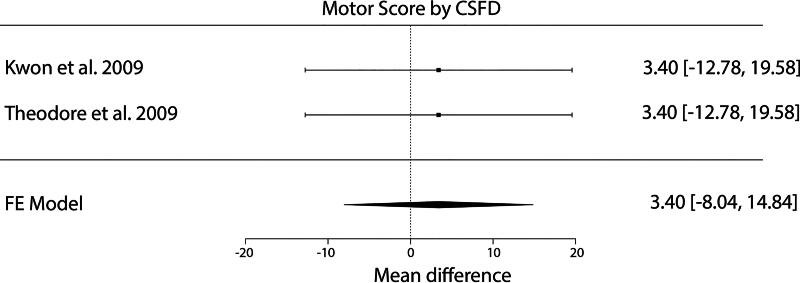
Forest plot of mean difference in motor score by CSFD. In Cohort B, CSFD was not associated with a significant improvement in motor score (*p* = 0.560). CSFD, cerebrospinal fluid drainage.

A meta-analysis of the mean change in ISNCSCI motor scores showed no significant difference between patients in the CSFD and no CSFD groups (*p* = 0.560, 95% confidence interval [CI]: −8.0 to 14.8). A meta-regression controlling for age similarly showed no significant effect of CSFD on the mean change in ISNCSCI motor scores (*p* = 0.993) (Fig. 2).

**FIG. 3.**
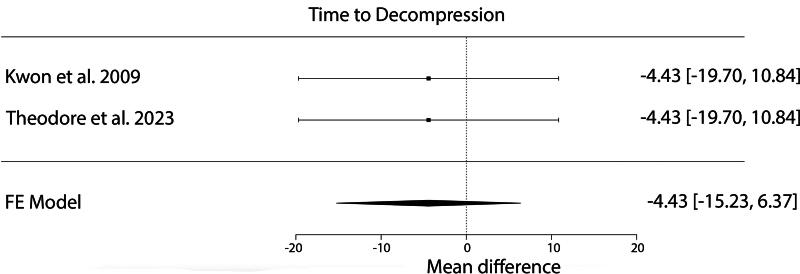
Forest plot of mean difference in motor score by time to decompression. There was no significant difference in motor score between a time to decompression of ≤19 h after injury relative to >19 h after injury (*p* = 0.421).

No significant difference in mean change in ISNCSCI motor scores was observed based on time to decompression (≤19 or >19 h after injury; *p* = 0.421, 95% CI: −15.2 to 6.4). When controlling for age, a meta-regression of the mean change in ISNCSCI motor scores showed no significant effect of time to decompression (*p* = 0.990) (Fig. 3).

## Discussion

The 2013 revision of the acute cervical SCI guidelines recommends maintaining an MAP of 85–90 mmHg for 1 week after injury.^22^ However, recent studies have suggested that MAP maintenance at ≥85 mmHg for 5 days after injury is associated with higher rates of short-term (but not long-term) neurological improvement,^23^ and others have reported limited benefits of vasopressor-induced MAP maintenance.^24,25^ Additionally, the use of vasopressors has been associated with possible complications such as myocardial infarction, arrhythmia, stroke, hypertensive hemorrhage, and posterior reversible encephalopathy syndrome.^26–31^ The 2024 guidelines weakly recommend MAP augmentation to a minimum of 75–80 mmHg, not to exceed 90–95 mmHg, for 3–7 days to optimize SCPP.^6^ However, the evidence remains limited in support of the use of LDs to reduce CSFP and improve spinal cord perfusion in tSCI.

We conducted a systematic review of the available evidence on the use of lumbar CSFD to improve neurological outcomes in acute tSCI. Cohort A showed no significant improvement in ASIA grade after lumbar CSFD. In Cohort B, a moderately significant improvement in ISNCSCI motor scores was seen in female patients and those who underwent an anterior approach to decompression (compared with a combined approach). There were no other significant indicators of improved motor scores in either cohort.

### Cohort A

There was no significant improvement in ASIA grade at the last follow-up visit among patients in Cohort A. Given the relatively small cohort of 13 patients, the analysis was likely underpowered to show the true effect of CSFD on motor function. The authors set a drainage limit of 10 mL at one time, with a maximum drainage of 30 mL over 24 h, and reported multiple instances in which no CSF could be drained. The resulting minimal fluid drainage was likely insufficient to significantly reduce ISP.^19^ Notably, in a more recent publication, Lavadi and colleagues^32^ found that empirical CSFD (5–10 mL per hour) had a lower risk of critical hypoperfusion than reactive drainage (restricted to SCPP <65 mmHg) for acute tSCI, with no significant difference in average SCPP or MAP. Although the study was limited by a small sample size, the findings suggest that empirical drainage with a 5- to 10-mL hourly rate is sufficient to reduce CSFP. Considering that the optimal SCPP significantly varies among patients,^10^ CSFD at an individualized hourly rate with MAP and SCPP monitoring may provide the greatest benefit for tSCI.

### Cohort B

After meta-analysis, we found no significant effect of CSFD on ISNCSCI motor score improvement, despite similar baseline neurological function between the groups. With only 33 cases, our analysis may have been underpowered to demonstrate any neurological benefit of CSFD. More aggressive CSFD may also be necessary to significantly improve motor scores. The study by Kwon et al.^20^ had a conservative protocol with a drain limit of 10 mL per hour, no target CSFP, and no drainage during periods when a neurological exam could not be obtained, which resulted in minimal CSFD for some patients. Considering that these patients made up 66.7% of our Cohort B, the conservative CSFD and lack of ISP goals may have contributed to our negative results. Theodore et al.^3^ set MAP goals and a target CSFP of <10 mmHg (similar to CSFP 10-mmHg targets reported in cardiovascular studies),^11,33^ with no other restrictions on drainage, allowing for sufficient CSF release to optimize SCPP. However, the 11 patients in this study made up only 33.3% of Cohort B, which may underrepresent the benefit of CSFD. Many studies have shown that CSFD during open and endovascular thoracoabdominal aortic aneurysm repair may reduce paraplegia^11–14,17,34^ and improve long-term survival in a subset of patients.^12^ Negative studies indicate that a sufficient volume of CSF must be drained to reduce ISP and show neurological benefit.^35^ With an optimized approach to SCPP management and monitoring, CSFD may improve neurological outcomes in patients with acute tSCI. However, large-scale randomized studies are needed to accurately assess the role of CSFD for acute tSCI.

In the combined cohort, female sex was associated with a significantly greater improvement in ISNCSCI motor score after LD placement relative to male sex. This may be related to lower levels of estrogen in men, as estrogen has demonstrated neuroprotective properties in central nervous system injury.^36–38^ In a multi-institutional retrospective study of differences in SCI between the sexes, Sipski et al.^36^ found that women with complete and incomplete SCI had significantly greater improvement in ASIA motor scores at 1 year following admission relative to men. Large-scale prospective studies are needed to validate these results and assess whether sex is a true predictor of motor outcome after tSCI.

On multivariate analysis, we found a significant improvement in ISNCSCI motor scores of patients who underwent anterior decompression compared with those who underwent combined decompression. Although combined anterior and posterior fusion results in improved stability, there is a greater risk of surgical trauma and complications with a combined approach relative to isolated anterior or posterior decompression.^39,40^ Additional uncontrolled factors may also influence this finding, including the level of injury and the severity of injury.

We found no significant difference in ISNCSCI motor score improvement between early and late decompressive surgery. The literature is mixed regarding the optimal time from injury to decompression in tSCI. Fehlings et al.^41^ and Dakson et al.^23^ reported that decompression within 24 h of tSCI is associated with greater neurological improvement than surgery ≥24 h after injury, with follow-up periods of 6 and 8 months, respectively. In a retrospective analysis of 73 patients with cervical SCI, Park et al.^42^ found that time from injury to decompression did not significantly affect neurological outcomes. Middendorp and colleagues^43^ performed a meta-analysis in 2013 and found that early decompression (within 24 h of injury in 10 studies and within 72 h in 6 studies) resulted in better motor scores and a higher rate of neurological improvement, but there was significant heterogeneity among studies. More recent literature has explored the role of “ultra-early” decompression (≤8–12 h after injury), but there is currently insufficient evidence to support its utility, and “ultra-early” decompression may be limited by delays in hospital transit and time to the operating room.^44^ Despite variability in the data, the literature is generally supportive of decompression within 24 h of injury,^45^ and the current AO Spine/Praxis guidelines recommend decompression ≤24 h of tSCI for adult patients regardless of injury severity and level.^46,47^

Spinal cord pressure may be monitored at the site of injury using a probe inserted during decompression (ISP) or obtained through a monitor in the lumbar cistern (CSFP).^9^ The optimal location for pressure measurement in acute SCI is a matter of debate because a pressure differential exists across the injury site. In SCI, the edematous cord expands against the nonelastic dura, resulting in a greater pressure at the level of injury relative to CSFP cranially or caudally.^7,9,19^ Severe edema may displace CSF from the site of injury, preventing sufficient fluid sampling if CSFD is attempted at the same level.^19,48^ In the case of cord injury, subdural ISP measured at the injury site closely approximates intraparenchymal ISP, supporting subdural probe placement, which is significantly less invasive.^8,9,49–51^ In the absence of significant cord edema or subdural occlusion, CSFP recorded at the lumbar cistern is comparable with ISP at the injury site.^9,51^ This highlights the clinical value of decompression and the potential benefit of expanded duraplasty,^9^ although these operations result in changes in ISP and CSFP that should be considered for optimal SCPP management. Kwon and colleagues^20^ noted a paradoxical increase in CSFP after surgical decompression^52^ and a subsequent increase in CSFP during postoperative lumbar catheter monitoring in select patients, suggesting that, with constant MAP, cord perfusion may transiently decrease in the immediate postoperative period.^20^ Cord edema after injury differs significantly among patients and evolves over time,^7^ which may contribute to the high variability in ISP noted in prior literature.^53^ The optimal SCPP for autoregulation also varies among patients.^10^ Anticipating CSFP changes after injury and after decompression may allow for effective CSFD and SCPP optimization. Considering the reported fluctuation in ISP^19^ and the variability in ISP/CSFP and SCPP among patients, a focus on individualized management of SCPP may improve outcomes.^48^

As a systematic review and meta-analysis, our study has inherent limitations, including possible selection or publication bias and heterogeneity due to variations in study design, patient demographics, and reporting outcomes, which may introduce potential bias. In particular, the lack of uniformity in the choice of functional grading scales and reported outcomes significantly limited the eligible studies. The more conservative drainage protocol by Kwon and colleagues^20^ may have also influenced the observed treatment effect in Cohort B. We controlled for age in both meta-regressions but were unable to control for other factors, such as sex, level of injury, and duration of an indwelling LD. Because of the relative paucity of published literature on the benefit of LDs and CSFD in tSCI, only 3 studies met the inclusion criteria, and a meta-analysis was only performed on the 2 studies that reported follow-up motor scores.^19^ Thus, our cohort is relatively small, and our analysis may be underpowered to show a significant treatment effect. To account for this, we used individual participant data to improve the statistical power, better identify inconsistencies in the data, and perform more detailed analyses. As a result of the small sample size, although the multivariate linear regression used to compare the mean difference in motor score for sex and approach was significant, the overall model only approached statistical significance (*F* = 2.16, *p* = 0.08). For this reason, the findings for sex and approach should be interpreted with caution and require validation by future studies. In addition, the available literature lacks sufficient follow-up periods. Cohort A had a mean follow-up of just 6 months,^19^ and only 64% of patients completed the 180-day follow-up in one study included in Cohort B.^3^ SCI is a heterogeneous pathology and cannot be fully characterized by ISNCSCI or ASIA motor scores. To better evaluate SCI interventions, we must first identify novel predictive biomarkers, both molecular and radiographic. Additionally, although lumbar catheters are widely available, CSFP/ISP monitoring is not universally performed, and SCPP management is currently limited to specialized centers across the United States.^6^ Large-scale randomized trials are needed to assess the role of lumbar CSFD to improve neurological outcomes in tSCI and develop recommendations to optimize SCPP. The multicenter, prospective, observational patient registry Transforming Research and Clinical Knowledge in SCI is currently enrolling patients to create a robust database for tSCI.^54^ The Spinal Cord Perfusion Pressure and Biomarker Study (NCT03911492) is also currently underway and will assess the neurological benefit of maintaining SCPP ≥65 mmHg in acute SCI and identify CSF biomarkers for neurological recovery. The study may provide elucidation on the role of SCPP maintenance, MAP augmentation, and CSFD in acute SCI.

## Conclusion

Lumbar CSFD and time from injury to decompression were not associated with neurological recovery after acute tSCI. Female sex was associated with a greater improvement in ISNCSCI motor score relative to male sex, and anterior decompression was associated with greater neurological recovery relative to the combined approach. CSFD with a target CSFP rather than volume restrictions for drainage may allow for sufficient fluid drainage to reduce ISP and maximize SCPP. Because of the interpatient and chronological variability in CSFP, patients may benefit most from individualized management of SCPP. Additional research currently underway may help to establish clear recommendations for the monitoring and management of SCPP.

## Transparency, Rigor, and Reproducibility Statement

This review was not registered by PROSPERO. A systematic review was performed according to PRISMA guidelines. A search was performed in July 2024 to identify relevant publications from five databases (PubMed, MEDLINE, Scopus, Web of Science, and Cochrane). The Covidence systematic review software (Veritas Health Innovation, Melbourne, Australia) was used to select articles that met the following criteria: (1) acute tSCI; (2) placement of lumbar catheter with CSFD; and (3) available ASIA grade or ISNCSCI motor scores. Included studies were divided into two cohorts according to the availability of ISNCSCI data. All statistical analyses were performed in R (v2023.06, R Foundation for Statistical Computing, Vienna, Austria). Descriptive statistics were calculated for Cohort A, and Wilcoxon signed rank tests were used to compare baseline and follow-up ASIA grades. For Cohort B, a meta-analysis was performed comparing the mean change in ISNCSCI motor scores at baseline and at the last follow-up by CSFD group. A separate meta-analysis of the mean change in motor scores was conducted for a time to decompression of <19 h versus ≥19 h, an interval chosen based on the median time to decompression in Cohort B. A fixed-effects model was used because of minimal heterogeneity between the studies. The effect of indwelling LD duration on neurological function was assessed using the nonparametric Mann–Whitney U test. A multivariate linear regression was performed to identify factors predictive of an improvement in motor score. We did not include a funnel plot for the assessment of reporting bias due to the limited number of studies included in the analysis. This article will be published under a Creative Commons Open Access license, and upon publication will be freely available at https://home.liebertpub.com/publications.

## Abbreviations Used

ASIA: American Spinal Injury Association
CSFD: cerebrospinal fluid drainage
CSFP: cerebrospinal fluid pressure
ISNCSCI: International Standards for Neurological Classification of Spinal Cord Injury
ISP: intraspinal pressure
LD: lumbar drain
MAP: mean arterial pressure
PRISMA: Preferred Reporting Items for Systematic Review and Meta-analyses
RCT: randomized controlled trial
SCPP: spinal cord perfusion pressure
tSCI: traumatic spinal cord injury

## References

[1] GBD Traumatic Brain Injury Spinal Cord Injury Collaborators. Global, regional, and national burden of traumatic brain injury and spinal cord injury, 1990–2016: A systematic analysis for the Global Burden of Disease Study 2016. Lancet Neurol 2019;18(1):56–87; doi: 10.1016/S1474-4422(18)30415-0 30497965 PMC6291456

[2] Ahuja CS, Wilson JR, Nori S, et al. Traumatic spinal cord injury. Nat Rev Dis Primers 2017;3:17018; doi: 10.1038/nrdp.2017.18 28447605

[3] Theodore N, Martirosyan N, Hersh AM, et al. Cerebrospinal fluid drainage in patients with acute spinal cord injury: A multi-center randomized controlled trial. World Neurosurg 2023;177:e472–e479; doi: 10.1016/j.wneu.2023.06.078 37356491

[4] Evaniew N, Davies B, Farahbakhsh F, et al. Interventions to optimize spinal cord perfusion in patients with acute traumatic spinal cord injury: An updated systematic review. Global Spine J 2024;14(3_suppl):58S–79S; doi: 10.1177/21925682231218737 38526931 PMC10964891

[5] Tator CH, Fehlings MG. Review of the secondary injury theory of acute spinal cord trauma with emphasis on vascular mechanisms. J Neurosurg 1991;75(1):15–26; doi: 10.3171/jns.1991.75.1.0015 2045903

[6] Kwon BK, Tetreault LA, Martin AR, et al. A clinical practice guideline for the management of patients with acute spinal cord injury: Recommendations on hemodynamic management. Global Spine J 2024;14(3_suppl):187S–211S; doi: 10.1177/21925682231202348 38526923 PMC10964888

[7] Squair JW, Belanger LM, Tsang A, et al. Spinal cord perfusion pressure predicts neurologic recovery in acute spinal cord injury. Neurology 2017;89(16):1660–1667; doi: 10.1212/WNL.0000000000004519 28916535

[8] Werndle MC, Saadoun S, Phang I, et al. Monitoring of spinal cord perfusion pressure in acute spinal cord injury: initial findings of the injured spinal cord pressure evaluation study*. Crit Care Med 2014;42(3):646–655. ; doi: 10.1097/CCM.0000000000000028 24231762

[9] Gee CM, Kwon BK. Significance of spinal cord perfusion pressure following spinal cord injury: A systematic scoping review. J Clin Orthop Trauma 2022;34:102024; doi: 10.1016/j.jcot.2022.102024 36147378 PMC9486559

[10] Saadoun S, Chen S, Papadopoulos MC. Intraspinal pressure and spinal cord perfusion pressure predict neurological outcome after traumatic spinal cord injury. J Neurol Neurosurg Psychiatry 2017;88(5):452–453; doi: 10.1136/jnnp-2016-314600 27864426

[11] Coselli JS, LeMaire SA, Koksoy C, et al. Cerebrospinal fluid drainage reduces paraplegia after thoracoabdominal aortic aneurysm repair: Results of a randomized clinical trial. J Vasc Surg 2002;35(4):631–639; doi: 10.1067/mva.2002.122024 11932655

[12] Safi HJ, Miller CC, 3rd, Huynh TT, et al. Distal aortic perfusion and cerebrospinal fluid drainage for thoracoabdominal and descending thoracic aortic repair: Ten years of organ protection. Ann Surg 2003;238(3):372–380; discussion 380-81; doi: 10.1097/01.sla.0000086664.90571.7a 14501503 PMC1422700

[13] Cina CS, Abouzahr L, Arena GO, et al. Cerebrospinal fluid drainage to prevent paraplegia during thoracic and thoracoabdominal aortic aneurysm surgery: A systematic review and meta-analysis. J Vasc Surg 2004;40(1):36–44; doi: 10.1016/j.jvs.2004.03.017 15218460

[14] Fedorow CA, Moon MC, Mutch WA, et al. Lumbar cerebrospinal fluid drainage for thoracoabdominal aortic surgery: Rationale and practical considerations for management. Anesth Analg 2010;111(1):46–58; doi: 10.1213/ANE.0b013e3181ddddd6 20522706

[15] Cheung AT, Pochettino A, McGarvey ML, et al. Strategies to manage paraplegia risk after endovascular stent repair of descending thoracic aortic aneurysms. Ann Thorac Surg 2005;80(4):1280–1288; discussion 1288-89; doi: 10.1016/j.athoracsur.2005.04.027 16181855

[16] Weigang E, Hartert M, Siegenthaler MP, et al. Perioperative management to improve neurologic outcome in thoracic or thoracoabdominal aortic stent-grafting. Ann Thorac Surg 2006;82(5):1679–1687; doi: 10.1016/j.athoracsur.2006.05.037 17062227

[17] Grant RA, Quon JL, Abbed KM. Management of acute traumatic spinal cord injury. Curr Treat Options Neurol 2015;17(2):334; doi: 10.1007/s11940-014-0334-1 25630995

[18] Page MJ, McKenzie JE, Bossuyt PM, et al. The PRISMA 2020 statement: An updated guideline for reporting systematic reviews. BMJ 2021;372:n71; doi: 10.1136/bmj.n71 33782057 PMC8005924

[19] Hogg FRA, Gallagher MJ, Kearney S, et al. Acute spinal cord injury: Monitoring lumbar cerebrospinal fluid provides limited information about the injury site. J Neurotrauma 2020;37(9):1156–1164; doi: 10.1089/neu.2019.6789 32024422

[20] Kwon BK, Curt A, Belanger LM, et al. Intrathecal pressure monitoring and cerebrospinal fluid drainage in acute spinal cord injury: A prospective randomized trial. J Neurosurg Spine 2009;10(3):181–193; doi: 10.3171/2008.10.SPINE08217 19320576

[21] RStudio. Integrated Development for R. 2020. Available from: http://www.rstudio.com/

[22] Walters BC, Hadley MN, Hurlbert RJ, et al. Guidelines for the management of acute cervical spine and spinal cord injuries: 2013 update. Neurosurgery 2013;60(CN_suppl_1):82–91. ; doi: 10.1227/01.neu.0000430319.32247.7f 23839357

[23] Dakson A, Brandman D, Thibault-Halman G, et al. Optimization of the mean arterial pressure and timing of surgical decompression in traumatic spinal cord injury: A retrospective study. Spinal Cord 2017;55(11):1033–1038; doi: 10.1038/sc.2017.52 28631747

[24] Weinberg JA, Farber SH, Kalamchi LD, et al. Mean arterial pressure maintenance following spinal cord injury: Does meeting the target matter? J Trauma Acute Care Surg 2021;90(1):97–106; doi: 10.1097/TA.0000000000002953 33003016

[25] Tsehay Y, Weber-Levine C, Kim T, et al. Advances in monitoring for acute spinal cord injury: A narrative review of current literature. Spine J 2022;22(8):1372–1387; doi: 10.1016/j.spinee.2022.03.012 35351667

[26] Inoue T, Manley GT, Patel N, et al. Medical and surgical management after spinal cord injury: Vasopressor usage, early surgerys, and complications. J Neurotrauma 2014;31(3):284–291; doi: 10.1089/neu.2013.3061 24020382

[27] Readdy WJ, Whetstone WD, Ferguson AR, et al. Complications and outcomes of vasopressor usage in acute traumatic central cord syndrome. J Neurosurg Spine 2015;23(5):574–580; doi: 10.3171/2015.2.SPINE14746 26230417

[28] Evaniew N, Mazlouman SJ, Belley-Cote EP, et al. Interventions to optimize spinal cord perfusion in patients with acute traumatic spinal cord injuries: A systematic review. J Neurotrauma 2020;37(9):1127–1139; doi: 10.1089/neu.2019.6844 32024432

[29] Yue JK, Tsolinas RE, Burke JF, et al. Vasopressor support in managing acute spinal cord injury: Current knowledge. J Neurosurg Sci 2019;63(3):308–317; doi: 10.23736/S0390-5616.17.04003-6 28252264

[30] Readdy WJ, Saigal R, Whetstone WD, et al. Failure of mean arterial pressure goals to improve outcomes following penetrating spinal cord injury. Neurosurgery 2016;79(5):708–714; doi: 10.1227/NEU.0000000000001249 27759678

[31] Agarwal N, Aabedi AA, Torres-Espin A, et al. Decision tree-based machine learning analysis of intraoperative vasopressor use to optimize neurological improvement in acute spinal cord injury. Neurosurg Focus 2022;52(4):E9; doi: 10.3171/2022.1.FOCUS21743 35364586

[32] Lavadi RS, Johnson BR, Chalif JI, et al. Comparing reactive versus empiric cerebrospinal fluid drainage strategies for spinal perfusion pressure optimization in patients with acute traumatic spinal cord injuries. J Clin Neurosci 2024;127:110757; doi: 10.1016/j.jocn.2024.110757 39059336

[33] Khan SN, Stansby G. Cerebrospinal fluid drainage for thoracic and thoracoabdominal aortic aneurysm surgery. Cochrane Database Syst Rev 2012;10(10):CD003635; doi: 10.1002/14651858.CD003635.pub3 23076900 PMC7173760

[34] Hnath JC, Mehta M, Taggert JB, et al. Strategies to improve spinal cord ischemia in endovascular thoracic aortic repair: Outcomes of a prospective cerebrospinal fluid drainage protocol. J Vasc Surg 2008;48(4):836–840; doi: 10.1016/j.jvs.2008.05.073 18723308

[35] Crawford E, Svensson LG, Hess KR, et al. A prospective randomized study of cerebrospinal fluid drainage to prevent paraplegia after high-risk surgery on the thoracoabdominal aorta. Journal of Vascular Surgery 1991;13(1):36–46; doi: 10.1016/0741-5214(91)90010-R 1987395

[36] Sipski ML, Jackson AB, Gomez-Marin O, et al. Effects of gender on neurologic and functional recovery after spinal cord injury. Arch Phys Med Rehabil 2004;85(11):1826–1836; doi: 10.1016/j.apmr.2004.04.031 15520978

[37] Elzer JG, Muhammad S, Wintermantel TM, et al. Neuronal estrogen receptor-alpha mediates neuroprotection by 17beta-estradiol. J Cereb Blood Flow Metab 2010;30(5):935–942; doi: 10.1038/jcbfm.2009.258 20010956 PMC2949189

[38] Brotfain E, Gruenbaum SE, Boyko M, et al. Neuroprotection by estrogen and progesterone in traumatic brain injury and spinal cord injury. Curr Neuropharmacol 2016;14(6):641–653; doi: 10.2174/1570159x14666160309123554 26955967 PMC4981744

[39] Ren C, Qin R, Wang P, et al. Comparison of anterior and posterior approaches for treatment of traumatic cervical dislocation combined with spinal cord injury: Minimum 10-year follow-up. Sci Rep 2020;10(1):10346; doi: 10.1038/s41598-020-67265-2 32587305 PMC7316727

[40] Ishak B, Abdul-Jabbar A, Glinski AV, et al. Comparing combined anterior and posterior to posterior-only decompression and fusion crossing the cervico-thoracic junction in octogenarians. Global Spine J 2023;13(1):164–171; doi: 10.1177/2192568221994793 33715487 PMC9837525

[41] Fehlings MG, Vaccaro A, Wilson JR, et al. Early versus delayed decompression for traumatic cervical spinal cord injury: Results of the Surgical Timing in Acute Spinal Cord Injury Study (STASCIS). PLoS One 2012;7(2):e32037; doi: 10.1371/journal.pone.0032037 22384132 PMC3285644

[42] Park JH, Kim JH, Roh SW, et al. Prognostic factor analysis after surgical decompression and stabilization for cervical spinal-cord injury. Br J Neurosurg 2017;31(2):194–198; doi: 10.1080/02688697.2016.1247781 27802777

[43] van Middendorp JJ, Hosman AJF, Doi SAR. The effects of the timing of spinal surgery after traumatic spinal cord injury: A systematic review and meta-analysis. J Neurotrauma 2013;30(21):1781–1794; doi: 10.1089/neu.2013.2932 23815524

[44] Adegeest CY, Moayeri N, Muijs SPJ, et al. Spinal cord injury: Current trends in acute management. Brain Spine 2024;4:102803; doi: 10.1016/j.bas.2024.102803 38618228 PMC11010802

[45] Wilson JR, Witiw CD, Badhiwala J, et al. Early surgery for traumatic spinal cord injury: Where are we now? Global Spine J 2020;10(1 Suppl):84S–91S; doi: 10.1177/2192568219877860 31934526 PMC6947677

[46] Tetreault LA, Kwon BK, Evaniew N, et al. A clinical practice guideline on the timing of surgical decompression and hemodynamic management of acute spinal cord injury and the prevention, diagnosis, and management of intraoperative spinal cord injury: Introduction, rationale, and scope. Global Spine J 2024;14(3_suppl):10S–24S; doi: 10.1177/21925682231183969 38632715 PMC10964894

[47] Fehlings MG, Tetreault LA, Hachem L, et al. An update of a clinical practice guideline for the management of patients with acute spinal cord injury: Recommendations on the role and timing of decompressive surgery. Global Spine J 2024;14(3_suppl):174S–186S; doi: 10.1177/21925682231181883 38526922 PMC10964895

[48] Phang I, Zoumprouli A, Saadoun S, et al. Safety profile and probe placement accuracy of intraspinal pressure monitoring for traumatic spinal cord injury: Injured Spinal Cord Pressure Evaluation study. J Neurosurg Spine 2016;25(3):398–405; doi: 10.3171/2016.1.SPINE151317 27129044

[49] Phang I, Papadopoulos MC. Intraspinal pressure monitoring in a patient with spinal cord injury reveals different intradural compartments: Injured Spinal Cord Pressure Evaluation (ISCoPE) study. Neurocrit Care 2015;23(3):414–418; doi: 10.1007/s12028-015-0153-6 26136148

[50] Werndle MC, Saadoun S, Phang I, et al. Measurement of intraspinal pressure after spinal cord injury: Technical note from the Injured Spinal Cord Pressure Evaluation study. Acta Neurochir Suppl 2016;122:323–328; doi: 10.1007/978-3-319-22533-3_64 27165930

[51] Saadoun S, Papadopoulos MC. Spinal cord injury: Is monitoring from the injury site the future? Crit Care 2016;20(1):308; doi: 10.1186/s13054-016-1490-3 27716379 PMC5050726

[52] Aarabi B, Olexa J, Chryssikos T, et al. Extent of spinal cord decompression in motor complete (American Spinal Injury Association Impairment Scale grades A and B) traumatic spinal cord injury patients: Post-operative magnetic resonance imaging analysis of standard operative approaches. J Neurotrauma 2019;36(6):862–876; doi: 10.1089/neu.2018.5834 30215287 PMC6484360

[53] Gee CM, Tsang A, Belanger LM, et al. All over the MAP: Describing pressure variability in acute spinal cord injury. Spinal Cord 2022;60(5):470–475; doi: 10.1038/s41393-022-00802-0 35418625

[54] Tsolinas RE, Burke JF, DiGiorgio AM, et al. Transforming Research and Clinical Knowledge in Spinal Cord Injury (TRACK-SCI): an overview of initial enrollment and demographics. Neurosurg Focus 2020;48(5):E6; doi: 10.3171/2020.2.FOCUS191030 32357323

